# 基于EMR-Lipid分散固相萃取-超高效液相色谱-串联质谱法同时测定牛肉中17种全氟烷基化合物

**DOI:** 10.3724/SP.J.1123.2024.02010

**Published:** 2024-11-08

**Authors:** Qiaoli QIU, Shengdong PAN, Li WANG, Lanyun FANG, Xiaohong CHEN, Micong JIN

**Affiliations:** 宁波市疾病预防控制中心, 浙江省微量有毒化学物健康风险评估技术研究重点实验室, 浙江 宁波 315010; Key Laboratory of Health Risk Appraisal for Trace Toxic Chemicals of Zhejiang Province, Ningbo Municipal Center for Disease Control and Prevention, Ningbo 315010, China

**Keywords:** 分散固相萃取, 超高效液相色谱-串联质谱, 全氟烷基化合物, 牛肉, dispersive solid-phase extraction (d-SPE), ultra performance liquid chromatography-tandem mass spectrometry (UPLC-MS/MS), perfluoroalkyl substances (PFASs), beef

## Abstract

建立了基于EMR-Lipid的分散固相萃取-超高效液相色谱-三重四极杆质谱法(UPLC-MS/MS)同时快速准确测定牛肉中17种全氟烷基化合物(perfluoroalkyl substances, PFASs)残留的检测方法。样品经含0.1%(v/v)甲酸的乙腈溶液提取后,采用100 mg PSA+80 mg C_18_+40 mg GCB+150 mg EMR-Lipid混合填料进行分散固相萃取净化,采用0.5 mmol/L氟化铵水溶液-甲醇体系为流动相,在Acquity Premier BEH C_18_色谱柱(100 mm×2.1 mm, 1.7 μm)上进行梯度洗脱分离,流速为0.3 mL/min,采用电喷雾负离子多反应监测(MRM)模式进行检测,内标法定量分析。在最佳的实验条件下,17种PFASs的线性范围为0.2~20.0 μg/L,相关系数为0.9915~0.9999。方法检出限为0.003~0.007 μg/kg,方法定量限为0.01~0.02 μg/kg。在0.05、0.5、1.8 μg/kg 3个加标水平下,17种PFASs的加标回收率为71.1%~127.4%, RSD为0.6%~14.4%。研究结果还表明:在本研究优化的色谱条件下,相比于不同浓度(2.0、5.0、10.0 mmol/L)的甲酸铵或乙酸铵水溶液-甲醇体系,0.5 mmol/L氟化铵水溶液-甲醇体系作为流动相时,17种PFASs的灵敏度均有提高,其中羧酸类长链PFASs(C_10_~C_18_)的灵敏度可提高1~2倍。相比于单独使用PSA、C_18_、GCB、EMR-Lipid吸附材料和PSA+C_18_+GCB组合吸附材料,本文选择的PSA+C_18_+GCB+EMR-Lipid混合吸附材料具有对油脂基质去除效果更佳的特点。该方法操作简便快速,灵敏度高,基质效应低,重复性好,可用于牛肉中多种全氟烷基化合物的同时快速准确检测。

## Abstract (en)

The ability to accurately analyze perfluoroalkyl substance (PFAS) levels in beef is imperative in order to effectively assess food-safety risks and ensure consumer safety because PFASs are harmful and prevalent in beef. In this study, we developed a rapid and accurate method for the simultaneously determination of the 17 PFASs in beef using dispersive solid-phase extraction (d-SPE) and ultra performance liquid chromatography-tandem mass spectrometry (UPLC-MS/MS), and optimized the mobile phase system, extraction solvent, and d-SPE materials. Samples were finally extracted using 0.1% (v/v) formic acid in acetonitrile, cleaned using d-SPE with PSA, C_18_, GCB, and EMR-Lipid, separated using an Acquity Premier BEH C_18_ column (100 mm×2.1 mm, 1.7 μm) with 0.5 mmol/L ammonium fluoride aqueous solution and methanol as the mobile phases at a flow rate of 0.3 mL/min. Analytes were detected in negative ion switching mode (ESI^-^) with multiple reaction monitoring (MRM) scanning, and quantitatively analyzed using the internal standard method. The 17 PFASs exhibited linearity in the 0.2-20.0 μg/L range under the optimal experimental conditions, with correlation coefficients of 0.9915-0.9999. The method delivered limits of detection (LODs) of 0.003-0.007 μg/kg and limits of quantification (LOQs) of 0.01-0.02 μg/kg. The 17 PFASs exhibited recoveries of 71.1%-127.4% with RSDs of 0.6%-14.4% when spiked at three levels (0.05, 0.5, and 1.8 μg/kg). We optimized the mobile phase system, which revealed that, compared with 2.0, 5.0, and 10.0 mmol/L ammonium formate or ammonium acetate in aqueous methanol, 0.5 mmol/L ammonium fluoride in aqueous methanol exhibited higher sensitivities for all the 17 PFASs, with PFASs bearing long-chain carboxylic acids (C_10_-C_18_) showing 1-2 fold increases in sensitivity. PFASs do not dissociate in acidic environments, favoring their entry into the organic phase. Therefore, we investigated the effect of extractant acidity, which revealed that the 17 PFASs were better extracted using 0.1% (v/v) formic acid in acetonitrile. The beef matrix has a complex composition; consequently, d-SPE adsorbents were required to purify samples and reduce matrix effects. The purification effects of four adsorbents (PSA, C_18_, GCB, and EMR-Lipid) toward the 17 PFASs and the amount of EMR-Lipid used were investigated, which revealed that 100 mg PSA+80 mg C_18_+40 mg GCB+150 mg EMR-Lipid exhibited superior matrix-purification behavior. We also investigated the effects of various injection solutions and types of syringe filter, with pure methanol selected for reconstitution and high-speed supernatant centrifugation applied prior to injection. The developed method is simple, rapid, sensitive, and reproducible, and can be used to simultaneously, rapidly, and accurately determine various perfluoroalkyl compounds in beef.

全氟烷基化合物(perfluoroalkyl substances, PFASs)是一类人工合成的新兴持久性有机化合物,因其具有较高的热稳定性及化学稳定性,同时具有极强的抗水解、抗光解、耐酸、耐碱能力^[1]^,已被广泛用于各种工业和生活产品的制造,PFASs通过工业排放以及日常生活用品的释放而进入整个生态系统,已有报道表明,在世界范围内的多种环境介质、食物,甚至母乳中均检出了全氟类化合物^[2,3]^,且能通过饮食摄入、皮肤接触、家居灰尘、空气吸入等途径在人体内积聚^[4]^。研究发现,PFASs具有器官毒性^[5]^、免疫毒性^[6,7]^、内分泌毒性^[8,9]^和生殖毒性^[10]^等,对人体健康产生危害。有研究显示,饮食是人类暴露于PFASs的重要途径,肉类又是PFASs饮食暴露的主要食物来源^[11]^。尽管已有文献报道了鱼肉、水产品中PFASs的检测方法^[12⇓⇓⇓⇓-17]^,但由于牛肉基质与鱼及水产品具有显著差异,方法的通用性不强。因此,建立一种简便、快速、灵敏度高的牛肉中PFASs的分析方法具有十分重要的现实意义。

液相色谱-串联质谱法(LC-MS/MS)是目前PFASs的主要检测方法之一,能够实现高通量快速检测,且假阳性率低,适用于痕量PFASs的检测分析^[12⇓⇓⇓⇓-17]^。由于肉类基质成分复杂,需采用预处理技术进行净化以减少基质效应,常用的净化方法有基于Oasis WAX和HLB 小柱的固相萃取(SPE)^[12,13]^、基于QuEChERS的分散固相萃取(d-SPE)^[14,15]^和基于m-PFC的滤过型固相萃取^[16,17]^等。SPE小柱需活化、上样、淋洗、洗脱等,步骤繁琐,且易堵塞,d-SPE净化法具有净化步骤少、成本低、溶剂使用量少、操作简便的优点^[15]^。目前市售的滤过型SPE小柱填料固定、种类单一,而d-SPE吸附剂的选择种类更多,更灵活,可以根据基质成分使用多种吸附材料达到理想的净化效果。已有报道的PFASs d-SPE吸附剂多为C_18_、石墨化炭黑(GCB)、*N*-丙基乙二胺(PSA)和多壁碳纳米管(MWCNTs)等^[12⇓⇓⇓⇓-17]^,较少见到增强型除脂材料EMR-Lipid d-SPE的应用。而EMR-Lipid具有对油脂等基质去除效果更佳的特点,在肉类食品的抗氧化剂、紫外吸收剂和阻燃剂等残留分析中已有应用^[18,19]^。同时由于PFASs的品种繁多,碳链长短不一,在PFASs的多组分残留同时检测时,色谱流动相改性剂的种类和浓度对PFASs的分析灵敏度也有显著影响,文献报道较多的有甲酸铵和乙酸铵^[12⇓⇓⇓⇓-17]^。已有研究证实在电喷雾负离子模式下,氟离子的存在能提高目标物的去离子化程度,如低浓度氟化铵可在电喷雾负离子模式下显著提高雌激素类化合物的信号响应值^[20,21]^。

本研究通过优化分散固相萃取填料和色谱流动相,建立了快速、准确、灵敏的基于EMR-Lipid混合填料的分散固相萃取-超高效液相色谱-三重四极杆质谱(UPLC-MS/MS)同时测定牛肉中17种PFASs的检测方法。

## 1 实验部分

### 1.1 仪器与试剂

Exion LC-TRIPLE QUAD 6500+超高效液相色谱-三重四极杆质谱仪(美国AB Sciex公司); Sigma 3-30K、Sigma 1-14K高速台式冷冻离心机(德国Sigma公司); N-EVAP 112氮吹仪(美国Organomation公司); Multi Reax振荡器(德国Heidolph公司); MJ-JC10绞肉机(中国美的公司); Solvs-UP3溶剂效应消除器(中国谱宁科技公司)。

乙腈、甲醇(HPLC级)购自美国ThermoFisher公司;甲酸铵、乙酸铵(HPLC级)购自德国Merck公司;甲酸、氟化铵(≥99.99%)购自麦克林公司;C_18_、GCB和PSA购自哈迈科技公司。PFASs混合标准品(全氟丁烷羧酸(PFBA)、全氟戊烷羧酸(PFPeA)、全氟丁烷磺酸(PFBS)、全氟己烷羧酸(PFHxA)、全氟庚烷羧酸(PFHpA)、全氟己烷磺酸(PFHxS)、全氟辛烷羧酸(PFOA)、全氟壬烷羧酸(PFNA)、全氟辛烷磺酸(PFOS)、全氟癸烷羧酸(PFDA)、全氟十一烷羧酸(PFUnDA)、全氟癸烷磺酸钠(PFDS)、全氟十二烷羧酸(PFDoDA)、全氟十三烷羧酸(PFTrDA)、全氟十四烷羧酸(PFTeDA)、全氟十六烷羧酸(PFHxDA)、全氟十八烷羧酸(PFODA), 5.0 μg/mL)和9种同位素混合内标(PFBA-^13^C_4_、PFHxA-^13^C_2_、PFOA-^13^C_4_、PFNA-^13^C_5_、PFDA-^13^C_2_、PFUnDA-^13^C_2_、PFDoDA-^13^C_2_、PFHxS-^18^O_2_、PFOS-^13^C_4_, 2 μg/mL)购自美国Wellington公司;Bond Elut EMR-Lipid d-SPE增强型除脂分散净化材料、含2 g无水MgSO_4_/NaCl (质量比为4∶1)的Bond Elut EMR-Lipid Polish萃取盐包购自美国Agilent公司。

40份牛肉样品购自当地菜市场和超市。

### 1.2 标准溶液配制

准确称取5.0 μg/mL17种PFASs混合标准品溶液100 μL至5.0 mL容量瓶中,用甲醇定容至刻度,配成质量浓度为100.0 μg/L的17种混合标准中间溶液,于-20 ℃避光保存。

准确移取100 μL混合内标溶液(2 μg/mL)至2 mL容量瓶中,用甲醇稀释至刻度,配成100 μg/L的9种PFASs混合内标工作液。

取适量17种混合标准中间溶液和混合内标工作溶液,用甲醇稀释定容,制得PFASs质量浓度分别为0.2、1.0、2.0、5.0、10.0、20.0 μg/L的系列混合标准溶液,其中内标的质量浓度均为5.0 μg/L。

### 1.3 样品前处理

准确称取2.0 g经充分绞碎的试样置于50 mL具塞离心管中,加入10.0 μL 100.0 ng/mL的17种PFASs混合内标工作液,准确加入3.0 mL超纯水,涡旋振荡3 min,加入7.0 mL含0.1%(v/v)甲酸的乙腈溶液,超声振荡15 min。然后加入100 mg PSA、80 mg C_18_、40 mg GCB和150 mg EMR-Lipid d-SPE净化材料,涡旋振荡5 min,再加入含2 g无水MgSO_4_/NaCl (质量比为4∶1)的萃取盐包,涡旋振荡2 min, 10000 r/min冷冻离心5 min,吸取上述全部上清液,氮吹至干。最后加入0.2 mL甲醇复溶,16000 r/min高速离心,移取上清液0.1 mL,供UPLC-MS/MS检测。

为了降低背景值,实验过程中应避免使用聚四氟乙烯材质的色谱管路与器皿。

### 1.4 色谱-质谱条件

色谱柱为Waters Acquity Premier BEH C_18_柱(100 mm×2.1 mm, 1.7 μm);流动相A为0.5 mmol/L氟化铵水溶液,流动相B为甲醇,流速为0.3 mL/min,柱温为40 ℃,进样体积为5 μL。梯度洗脱程序:0~2.0 min, 20%B~60%B; 2.0~10.0 min, 60%B~95%B; 10.0~12.0 min, 95%B; 12.0~12.1 min, 95%B~20%B; 12.1~15.0 min, 20%B。

检测方式:电喷雾电离,负离子模式(ESI^-^),多反应监测(MRM)。雾化器压力:344.7 kPa(50 psi),辅助器压力:344.7 kPa(50 psi),气帘气压力:275.8 kPa(40 psi),电喷雾电压为 -4500 V,离子源温度为500 ℃,其他的质谱参数见表1。

**表1:** 17种PFASs及9种内标的质谱参数

Compound	*t*_R_/ min	Precursor ion (*m/z*)	Product ion (*m/z*)	Declustering potential/V	Collision energy/eV	IS
Perfluorobutanoic acid (PFBA)	2.42	213.0	168.9^*^	-19	-13	PFBA-^13^C_4_
Perfluoropentanoic acid (PFPeA)	3.19	263.0	218.9^*^	-26	-11	PFBA-^13^C_4_
Perfluorobutanesulfonic acid (PFBS)	3.29	298.9	80.0^*^	-70	-65	PFOS-^13^C_4_
			99.0	-70	-36	
Perfluorohexanoic acid (PFHxA)	3.62	312.9	268.9^*^	-11	-11	PFHxA-^13^C_2_
			119.0	-11	-26	
Perfluoroheptanoic acid (PFHpA)	4.08	362.9	318.9^*^	-20	-14	PFHxA-^13^C_2_
			168.9	-20	-24	
Perfluorohexanesulfonic acid (PFHxS)	4.12	398.9	80.0^*^	-70	-75	PFHxS-^18^O_2_
			99.0	-70	-79	
Perfluorooctanoic acid (PFOA)	4.70	412.9	368.9^*^	-30	-15	PFOA-^13^C_4_
			168.9	-30	-25	
Perfluorononanoic acid (PFNA)	5.39	462.9	418.9^*^	-19	-14	PFNA-^13^C_5_
			218.9	-19	-23	
Perfluorooctanesulfonic acid (PFOS)	5.43	498.9	80.0^*^	-60	-96	PFOS-^13^C_4_
			99.0	-60	-99	
Perfluorodecanoic acid (PFDA)	6.09	512.9	468.9^*^	-33	-16	PFDA-^13^C_2_
			218.9	-33	-24	
Perfluoroundecanoic acid (PFUnDA)	6.73	562.9	518.9^*^	-29	-19	PFUnDA-^13^C_2_
			268.9	-29	-26	
Perfluorodecanesulfonic acid sodium (PFDS)	6.73	598.8	79.9^*^	-100	-100	PFOS-^13^C_4_
			98.9	-100	-100	
Perfluorododecanoic acid (PFDoDA)	7.32	612.8	569.0^*^	-20	-19	PFDoDA-^13^C_2_
			168.9	-20	-33	
Perfluorotridecanoic acid (PFTrDA)	7.84	662.8	618.8^*^	-40	-17	PFDoDA-^13^C_2_
			168.9	-40	-34	
Perfluorotetradecanoic acid (PFTeDA)	8.27	712.8	668.8^*^	-40	-17	PFDoDA-^13^C_2_
			168.9	-40	-39	
Perfluorohexadecanoic acid (PFHxDA)	9.01	812.9	768.9^*^	-30	-22	PFDoDA-^13^C_2_
			168.9	-30	-35	
Perfluorooctadecanoic acid (PFODA)	9.58	912.9	868.9^*^	-30	-22	PFDoDA-^13^C_2_
			218.9	-30	-35	
Perfluoro-*n*-[^13^C_4_]butanoic acid (PFBA-^13^C_4_)	2.42	216.9	171.9^*^	-19	-13	
Perfluoro-*n*-[1,2-^13^C_2_]hexanoic acid (PFHxA-^13^C_2_)	3.61	314.9	269.9^*^	-11	-11	
Perfluoro-*n*-[1,2,3,4-^13^C_4_]octanoic acid (PFOA-^13^C_4_)	4.70	416.9	372.0^*^	-30	-15	
Perfluoro-*n*-[1,2,3,4,5-^13^C_5_]nonanoic acid (PFNA-^13^C_5_)	5.39	467.9	422.9^*^	-19	-14	
Perfluoro-*n*-[1,2-^13^C_2_]decanoic acid (PFDA-^13^C_2_)	6.08	514.9	469.9^*^	-33	-24	
Perfluoro-*n*-[1,2-^13^C_2_]undecanoic acid (PFUnDA-^13^C_2_)	6.73	564.9	519.9^*^	-29	-15	
Perfluoro-*n*-[1,2-^13^C_2_]dodecanoic acid (PFDoDA-^13^C_2_)	7.32	614.8	569.9^*^	-20	-19	
Sodium perfluoro-1-hexane[^18^O_2_]sulfonate (PFHxS-^18^O_2_)	4.12	402.9	83.9^*^	-70	-75	
Sodium perfluoro-1-[1,2,3,4-^13^C_4_]octanesulfonate (PFOS-^13^C_4_)	5.42	502.9	79.9^*^	-60	-96	

* Quantitative ion.

## 2 结果与讨论

### 2.1 仪器条件优化

#### 2.1.1 质谱条件的优化

使用注射泵将100.0 μg/L的PFASs混合标准溶液以10 μL/min的流速直接注入质谱仪中,17种PFASs和9种内标化合物在负离子模式下全扫描的准分子离子峰[M-H]^-^响应较强。确定母离子后优化去簇电压,使母离子的响应值最高,再用子离子扫描模式确定各自的子离子,其中PFBA和PFPeA因结构稳定,只能分别找到*m/z*为168.9和218.9的子离子,作为定量离子,与文献[14]报道一致。最后再优化其碰撞能量,最终确定所有化合物的定量离子对和定性离子对以及对应的去簇电压和碰撞能量(表1)。

#### 2.1.2 色谱柱的选择

实验比较了常用的Waters HSS T3(100 mm×2.1 mm, 1.8 μm)、Waters BEH C_18_(100 mm×2.1 mm, 1.7 μm)以及Acquity Premier BEH C_18_(100 mm×2.1 mm, 1.7 μm)3种色谱柱。结果表明,应用HSS T3和BEH C_18_色谱柱时,PFHxDA和PFODA的色谱峰信号较低,而且两个化合物在HSS T3和BEH C_18_色谱柱上的质谱响应较Premier BEH C_18_色谱柱低50%左右。可能是Premier BEH C_18_色谱柱采用的高性能表面(HPS)技术(有机/无机杂化表面技术)能减少化合物与柱硬件表面间的相互作用造成的样品损失,从而提高化合物的回收率、灵敏度和重复性。所以,实验选择Premier BEH C_18_柱作为实验用色谱柱。

#### 2.1.3 色谱流动相的优化

本研究选用水-甲醇体系进行流动相的优化,着重考察了不同浓度及种类的流动相改性剂(甲酸铵、乙酸铵和氟化铵)对17种PFASs分离效果和灵敏度的影响。结果表明,PFASs在不同浓度(2、5、10 mmol/L)甲酸铵和乙酸铵水溶液-甲醇体系中的灵敏度没有明显差异,其中5 mmol/L的乙酸铵和2 mmol/L甲酸铵使17种PFASs的灵敏度相对较高。采用0.5 mmol/L氟化铵水溶液-甲醇体系,17种PFASs的灵敏度均有不同程度的提高,且羧酸类PFASs的提高幅度总体高于磺酸类PFASs,其中羧酸类长链PFASs(C_10_~C_18_)的灵敏度(以峰信号强度计)提高1~2倍,结果见图1。说明氟化铵可在电喷雾负离子模式下为PFASs提供更好的信号,这可能是在电喷雾离子化过程中,氟离子的存在导致长链PFASs的去质子化程度增强,即[M-H]^-^比其他的阴离子具有更高的丰度。10.0 μg/L的17种PFASs标准溶液的MRM色谱图见图2。

**图1:**
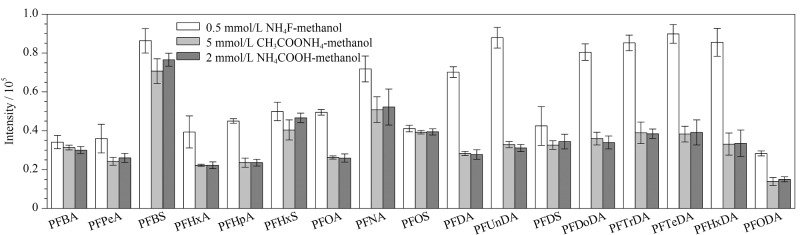
不同流动相体系对0.2 μg/L PFASs峰强度的影响(*n*=3)

**图2:**
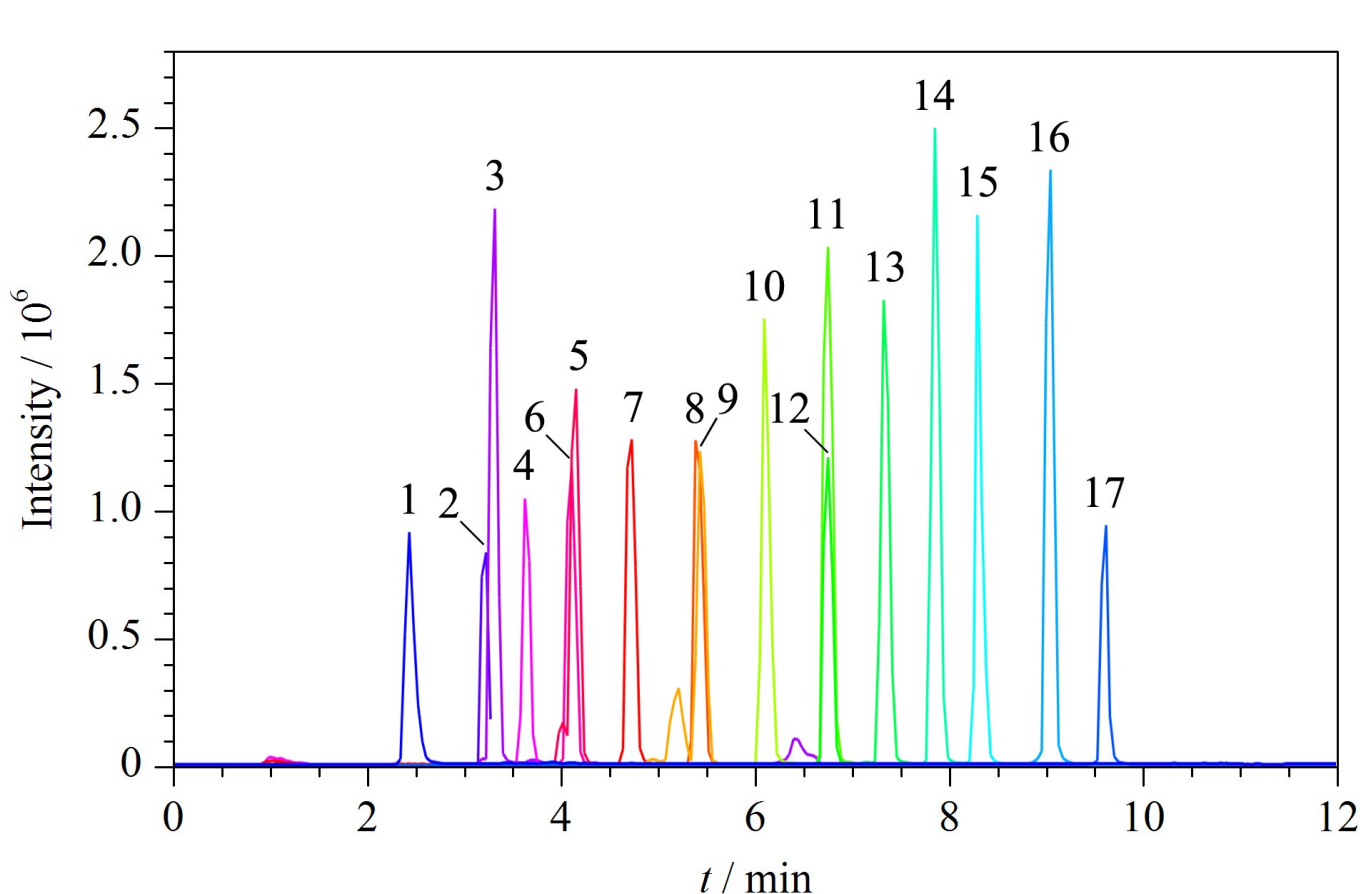
17种PFASs的MRM色谱图

### 2.2 前处理方法的优化

#### 2.2.1 提取溶剂优化

PFASs在酸性环境下呈非解离状态,有利于进入有机相,因此改变提取剂的酸度避免酸性目标物离子化,可适当提高提取效率^[15,16]^;文献[16]已证实随着溶液中甲酸体积分数的增加,PFASs的提取效率逐渐变低。因此,本文考察了含不同体积分数(0、0.05%、0.1%、0.2%、0.3%)甲酸的乙腈溶液对牛肉中17种PFASs峰面积的影响(见图3)。

**图3:**
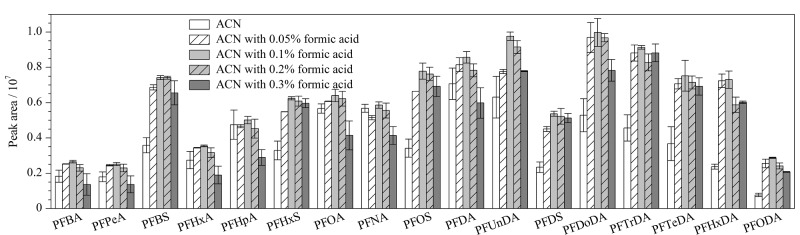
不同提取溶剂对17种PFASs峰面积的影响(*n*=3)

结果显示,随着甲酸体积分数的增加,峰面积先增加后降低。且采用含0.1%(v/v)甲酸的乙腈提取时,17种PFASs的峰面积最高,表明在合适的酸性条件下目标物更容易被有机溶剂提取。综上,选择含0.1%(v/v)甲酸的乙腈溶液作为提取溶剂。

#### 2.2.2 净化材料种类及用量的选择

分散固相萃取常用的吸附剂有C_18_、PSA和GCB, C_18_能吸附基质中脂质等弱极性共提取物;PSA能有效除去有机酸等物质;GCB能有效去除色素和甾醇类等干扰物^[14,15]^。文献[14,15]报道:在肉类产品中使用100 mg PSA、80 mg C_18_和30~40 mg GCB净化,对PFASs具有较好的回收率,但随着C_18_、PSA和GCB使用量的增加,对PFASs的吸附作用也增加,从而影响回收率。然而EMR-Lipid吸附材料具有对油脂等基质去除效果更佳的特点。因此,考虑使用EMR-Lipid新型高效除脂净化材料。本研究着重对EMR-Lipid的使用量(0、50、100、150、200、500、800 mg)进行优化(见图4)。在使用100 mg PSA、80 mg C_18_和40 mg GCB分散固相萃取填料的基础上,增加不同使用量的EMR-Lipid填料后,17种PFASs的峰面积均有提高,表明PSA、C_18_、GCB和EMR-Lipid组合净化牛肉中的17种PFASs效果更佳。这可能是因为EMR-Lipid有效去除了牛肉基质中的油脂,从而有效减少基质效应对目标分析物的影响。结果还显示,随着EMR-Lipid使用量的增加,17种PFASs的峰面积呈先上升后下降趋势,并在150 mg和200 mg时达到最大值。考虑到增加EMR-Lipid吸附剂用量可能会带来实验成本增加及杂质引入,最后确定使用量为150 mg。

**图4:**
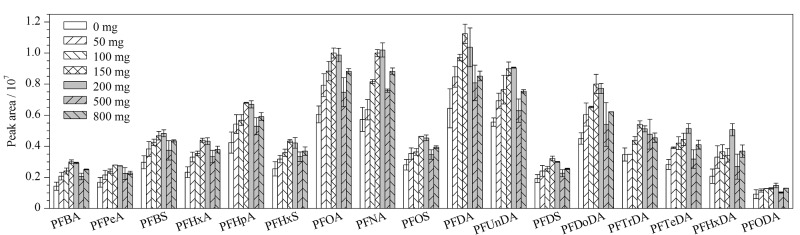
不同用量的EMR-Lipid填料对17种PFASs峰面积的影响(*n*=3)

#### 2.2.3 复溶溶剂的选择

实验比较了用甲醇-水(1∶4、1∶1、4∶1和1∶0, v/v)进行复溶的效果(见图5)。结果显示,甲醇-水(1∶4, v/v)几乎无法复溶17种PFASs,但随着甲醇比例的增加,PFASs的复溶量也逐渐升高,短链PFASs(C_4_~C_8_)在甲醇-水(4∶1, v/v)和甲醇中复溶量达到最大值且较为接近,长链PFASs(C_9_~C1_8_)在甲醇中复溶量最大。可见长链PFASs在水中的溶解性差,在甲醇中溶解性好。但用甲醇作为复溶剂,与甲醇-水(1∶4, v/v)的初始流动相不匹配,可能存在溶剂效应。实验结果显示,以甲醇为复溶溶剂时,除了PFBA和PFPeA峰形较差、延迟出峰外,其余PFASs不存在溶剂效应。为了改善PFBA和PFPeA的溶剂效应,本实验采用溶剂效应消除器,结果显示,PFBA和PFPeA的峰形和延迟出峰的情况得到明显改善,虽然PFBA的峰形还是存在一定的展宽(如图2),但不影响定量。因此,实验最后选择甲醇作为复溶溶剂。

**图5:**
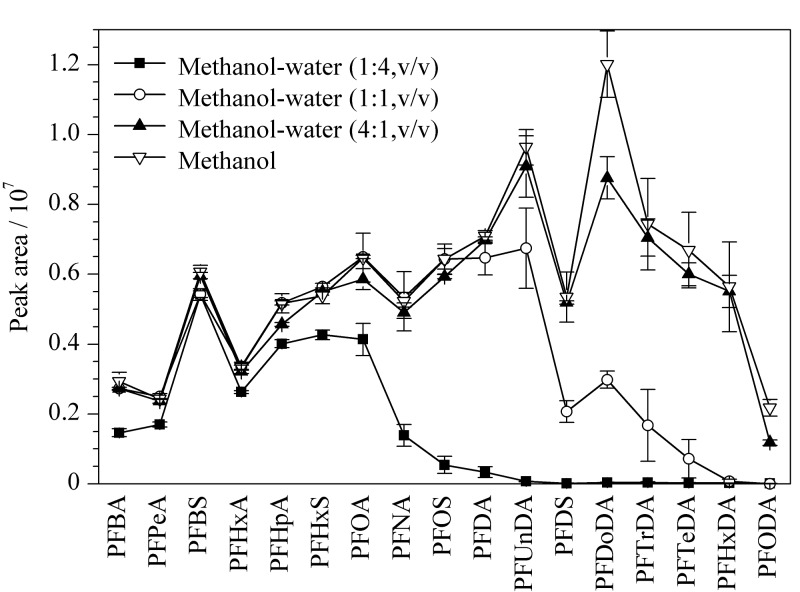
不同复溶溶剂对复溶效果的影响(*n*=3)

#### 2.2.4 进样液前处理方法优化

微孔针式滤器主要用于色谱分析中流动相及样品的过滤,对保护色谱柱、液相系统和质谱离子源等不被污染具有良好的作用。但是其使用前需进行考察,明确是否对待测成分有吸附作用、是否有溶出目标化合物。因此,本实验比较了各种不同材质(疏水聚四氟乙烯(hydrophobic PTFE)、疏水聚偏氟乙烯(PVDF)、尼龙(Nylon)、再生纤维素(RC))的针式滤器(均为13 mm×0.22 μm)本底溶出情况以及对待测化合物的吸附情况。实验结果显示,疏水PTFE和RC针式滤器无目标PFASs本底溶出,Nylon针式滤器有PFOA本底溶出,PVDF针式滤器有PFHxA、PFHpA、PFOA、PFNA和PFDA本底溶出。结果还显示不同材质针式滤器对PFHxDA和PFODA均有明显吸附(如图6),对其他PFASs基本无吸附。其中RC对PFHxDA和PFODA的吸附性最大,几乎全部吸附。疏水PTFE对PFODA和PFHxDA的吸附率分别为100%和48.9%, PVDF对PFODA和PFHxDA的吸附率分别为100%和30.2%, Nylon对PFODA的吸附率为10.3%,对PFHxDA几乎无吸附。考虑针式滤器对目标化合物有本底溶出和吸附影响,因而不选择用针式滤器过滤样品,而将样液16000 r/min冷冻离心5 min后取上清液待用。

**图6:**
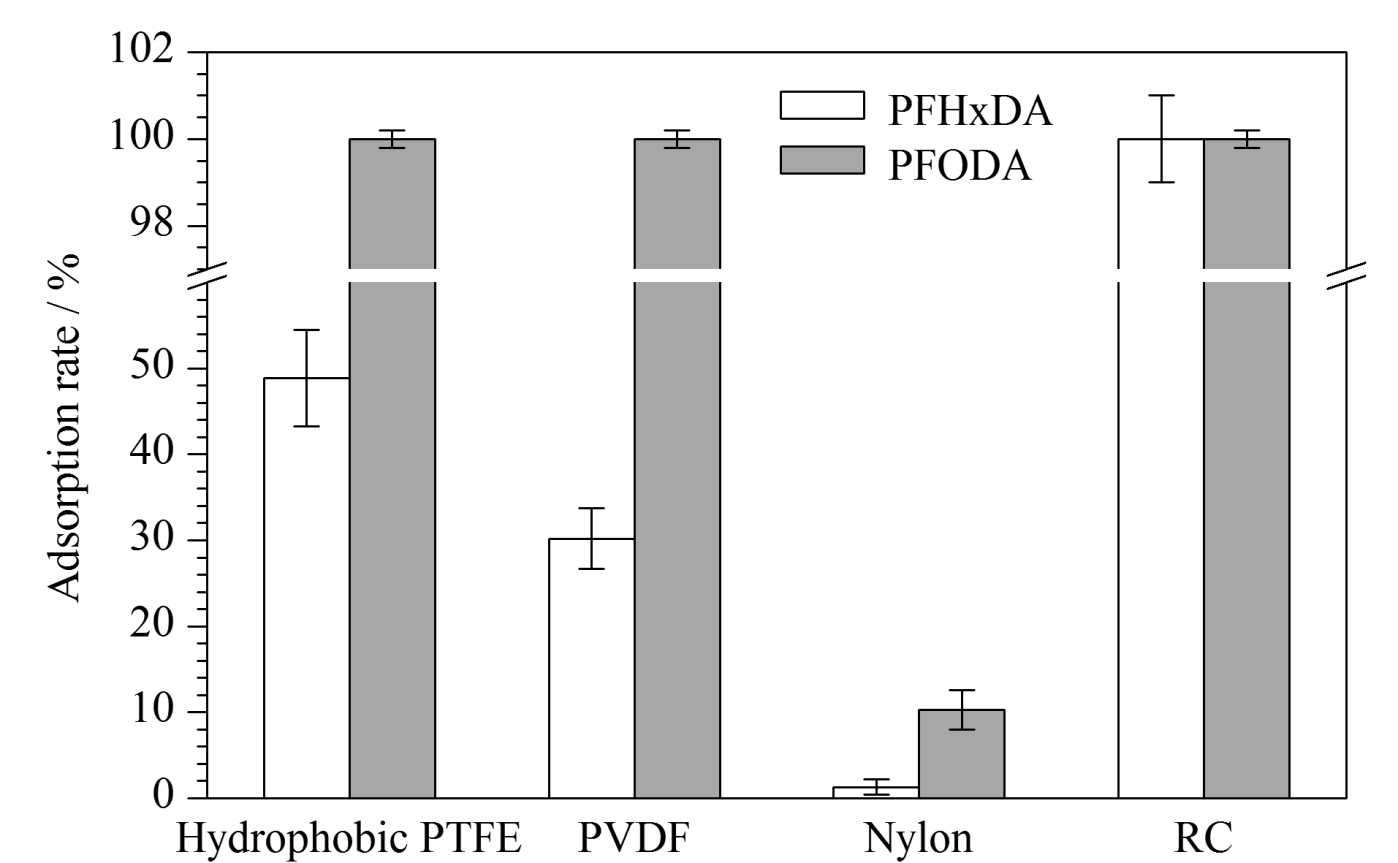
不同针式滤器的吸附率比较(*n*=3)

### 2.3 基质效应考察

基质效应(ME)=(基质匹配标准曲线斜率-溶剂标准曲线斜率)/溶剂标准曲线斜率×100%。ME>0,表明存在基质增强效应;ME<0,表明存在基质抑制效应。当│ME│<20%时,表示弱基质效应;当│ME│为20%~50%时,表示中等基质效应;当│ME│>50%时,表示强基质效应^[21]^。分别对系列溶剂标准溶液和相同质量浓度的系列基质匹配标准溶液进行UPLC-MS/MS检测,计算基质效应(见图7)。结果表明,13种PFASs表现为弱基质效应;PFBA、PFPeA、PFHxA和PFOA表现为中等基质效应。实验结果显示,样品经前处理后,PFASs的基质效应无法全部降低为弱基质效应,因此本实验采用内标法定量以降低基质效应对目标化合物定量测定的影响。

**图7:**
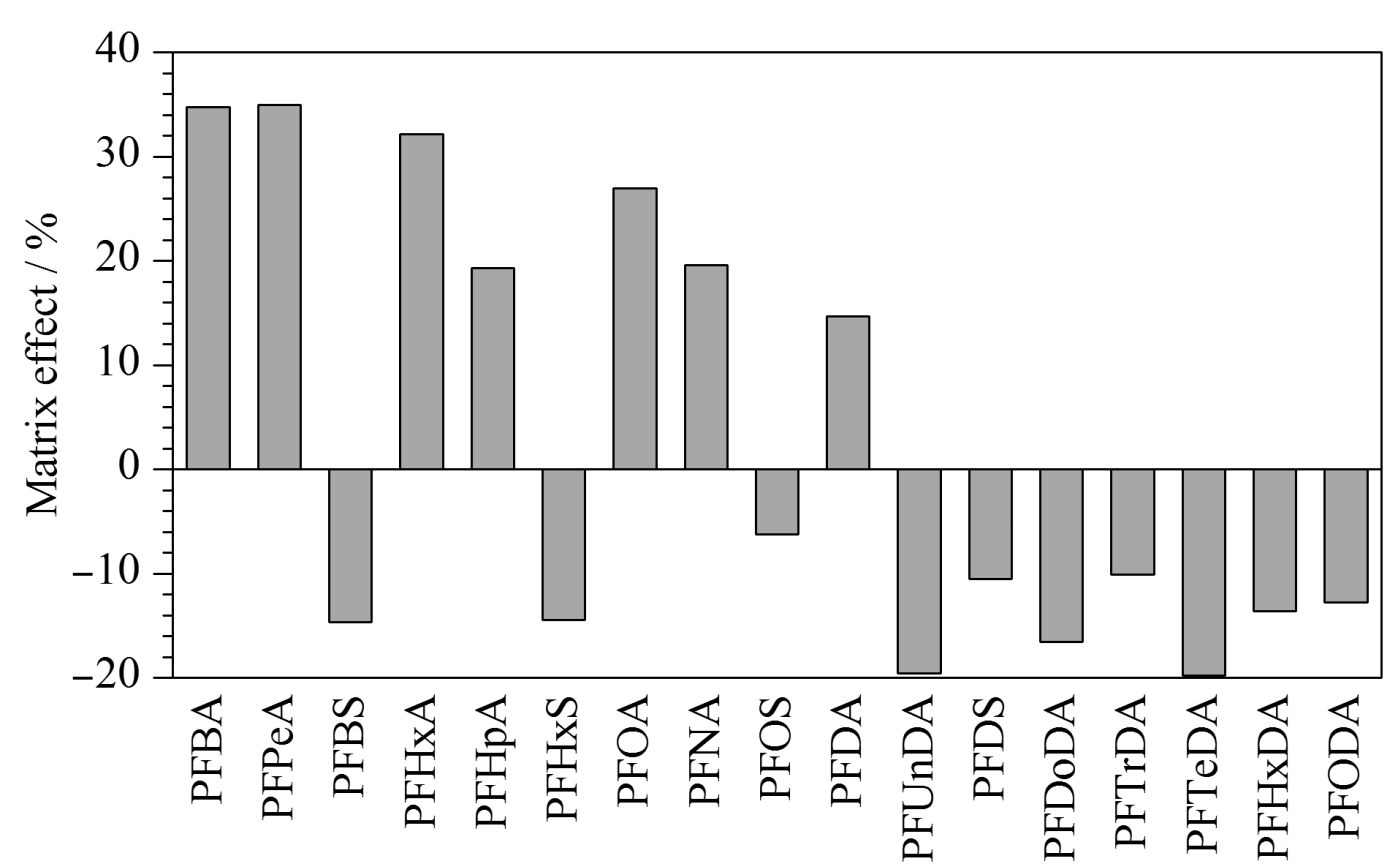
牛肉基质中17种PFASs的基质效应

### 2.4 方法学考察

#### 2.4.1 线性关系、相关系数、检出限和定量限

对配制的系列混合标准溶液进行分析,以各PFASs的峰面积与内标峰面积的比值为纵坐标,以PFASs的质量浓度与内标质量浓度的比值为横坐标,考察17种PFASs的线性关系。结果显示,17种PFASs在0.2~20.0 μg/L的范围内具有良好的线性关系,相关系数(*R*^2^)为0.9915~0.9999 (见表2)。采用空白样品加标的方式确定17种PFASs的方法检出限(*S/N*=3)为0.003~0.007 μg/kg,方法定量限(*S/N*=10)为0.01~0.02 μg/kg(见表2)。

**表2:** 17种PFASs的线性方程、相关系数、检出限和定量限

Compound	Linear equation	*R*^2^	LOD/ (μg/kg)	LOQ/ (μg/kg)
PFBA	*y*=1.00849*x*+0.0727	0.9994	0.007	0.02
PFPeA	*y*=0.99373*x*+0.1413	0.9985	0.007	0.02
PFBS	*y*=1.69526*x*-0.0103	0.9992	0.007	0.02
PFHxA	*y*=0.84395*x*+0.1736	0.9917	0.003	0.01
PFHpA	*y*=1.02703*x*+0.2281	0.9915	0.007	0.02
PFHxS	*y*=1.16612*x*-0.0007	0.9980	0.007	0.02
PFOA	*y*=1.06944*x*+0.2209	0.9995	0.003	0.01
PFNA	*y*=0.94917*x*+0.1736	0.9941	0.003	0.01
PFOS	*y*=1.23867*x*+0.0096	0.9999	0.007	0.02
PFDA	*y*=0.49088*x*+0.0597	0.9997	0.003	0.01
PFUnDA	*y*=0.95842*x*-0.0013	0.9981	0.003	0.01
PFDS	*y*=0.58787*x*-0.0118	0.9958	0.007	0.02
PFDoDA	*y*=0.72798*x*-0.0007	0.9998	0.003	0.01
PFTrDA	*y*=0.69375*x*-0.0025	0.9981	0.003	0.01
PFTeDA	*y*=0.70892*x*-0.0190	0.9959	0.003	0.01
PFHxDA	*y*=0.37967*x*-0.0156	0.9970	0.007	0.02
PFODA	*y*=0.44318*x*-0.0719	0.9994	0.007	0.02

*y*: peak area ratio of PFASs to internal standard; *x*: mass concentration ratio of PFASs to internal standard.

#### 2.4.2 加标回收率和精密度

以空白牛肉样品为基质,添加低(0.05 μg/kg)、中(0.5 μg/kg)、高(1.8 μg/kg) 3个水平的混合标准溶液,按1.3节步骤进行样品前处理,采用UPLC-MS/MS检测。同时做基质空白和过程空白实验,扣除实验本底值后计算加标回收率。每个加标水平做6次平行实验,结果显示,17种PFASs在低、中、高3个加标水平下的回收率分别为71.1%~127.4%、81.4%~123.6%、74.0%~120.1%(*n*=6), RSD分别为0.6%~14.4%、1.3%~9.3%、0.8%~12.9%(*n*=6),见表3。

**表3:** 17种PFASs的加标回收率和精密度(*n*=6)

Compound	0.05 μg/kg			0.5 μg/kg			1.8 μg/kg		
	Recovery/ %	RSD/ %		Recovery/ %	RSD/ %		Recovery/ %	RSD/ %	
PFBA	89.3	10.0		100.1	3.9		97.9	7.0	
PFPeA	88.9	9.1		81.4	2.5		83.9	9.7	
PFBS	104.0	4.6		103.7	9.3		105.2	6.8	
PFHxA	74.6	4.5		90.7	3.4		101.2	6.7	
PFHpA	127.1	5.7		123.6	4.8		116.9	0.8	
PFHxS	82.1	7.1		96.6	2.7		98.9	2.9	
PFOA	71.1	5.0		92.9	2.4		79.7	5.3	
PFNA	111.6	2.5		104.8	4.2		96.5	5.0	
PFOS	106.1	0.6		102.4	4.1		110.4	7.2	
PFDA	97.2	2.9		103.8	7.5		105.9	9.4	
PFUnDA	99.9	3.5		92.3	3.7		79.5	12.9	
PFDS	111.9	5.4		101.2	2.1		104.8	4.7	
PFDoDA	124.9	1.0		112.5	1.4		120.1	9.5	
PFTrDA	101.4	12.9		100.6	4.6		97.5	8.5	
PFTeDA	122.7	14.4		104.1	1.7		80.8	5.1	
PFHxDA	127.2	2.7		106.1	6.4		74.3	7.3	
PFODA	127.4	6.7		97.0	1.3		74.0	5.6	

### 2.5 方法对比

将本文建立的方法和文献方法进行了对比,结果见表4。相较于HLB和WAX等固相萃取小柱上样、活化、淋洗、洗脱等步骤繁琐,本方法操作简便、耗时短。相较于滤过型净化小柱填料固定、难以避免对部分目标化合物的吸附问题,d-SPE法可根据目标化合物的特性灵活选择吸附材料种类及用量,减少目标化合物的吸附损失,达到更佳的净化效果。特别是针对超长链PFHxDA和PFODA这两个化合物极易吸附的特性,采用的d-SPE前处理方法减少了对PFHxDA和PFODA的吸附,提高了检测灵敏度。同时,本研究采用氟化铵作为液相色谱流动相的缓冲盐,提高了长链PFASs的检测灵敏度;通过高速离心取复溶上清液的方法,既改进了PFHxDA和PFODA在针式滤器中极易吸附损失的问题,又减少了复溶液的使用量,提高了PFASs的复溶浓度及检测灵敏度。因此,本方法的检出限水平低于多个方法,具有良好的灵敏度,满足痕量分析要求。

**表4:** 本研究建立的检测方法与文献方法的比较

Samples	Buffer salt type	Purification method	LOD/(μg/kg)		Compound *M*_r_	Ref.
Fish	CH_3_COONH_4_	WAX SPE	0.06-	0.19	214-614	[12]
Aquatic products	CH_3_COONH_4_	QuEChERS (C_18_+GCB)	0.006-	0.02	214-914	[14]
Pork, beef, lamb	CH_3_COONH_4_	QuEChERS (C_18_+PSA+GCB)	0.02-	0.05	214-700	[15]
Crab meat	CH_3_COONH_4_	m-PFC filtered SPE	0.03-	0.15	214-714	[16]
Fish	CH_3_COONH_4_	m-PFC filtered SPE	0.015-	0.05	214-714	[17]
Beef	NH_4_F	d-SPE (C_18_+PSA+GCB+EMR-Lipid)	0.003-	0.007	214-914	this study

### 2.6 样品测定

应用本研究建立的方法对市售的40份牛肉样品中的17种PFASs进行检测。结果显示,PFASs检出率为12.5%。其中3份检出PFOA,含量为0.3~1.8 μg/kg; 1份检出PFOS,含量为1.2 μg/kg, 1份检出PFDA,含量为0.08 μg/kg。典型牛肉样品的MRM色谱图见图8。

**图8:**
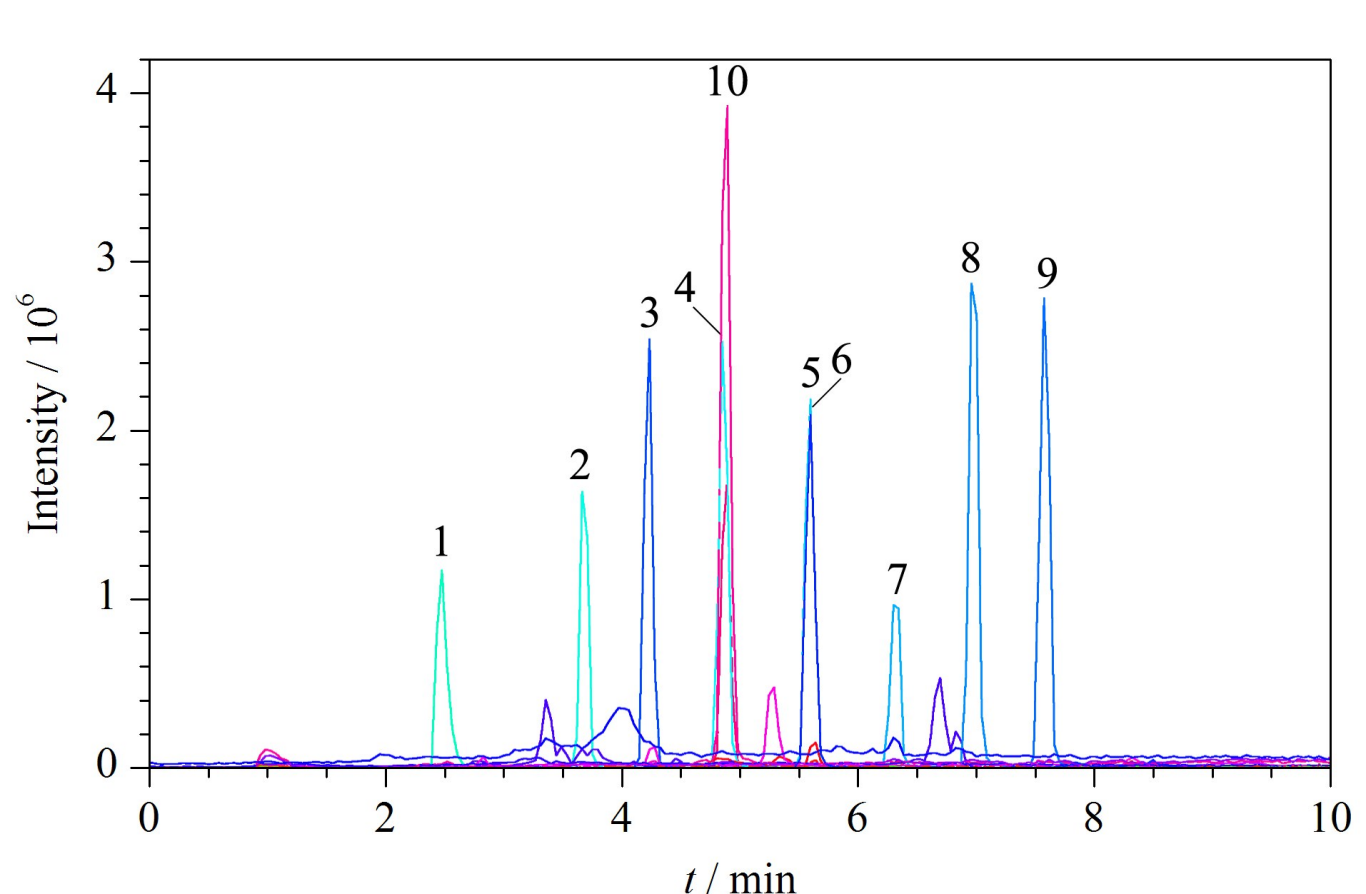
1份牛肉样品的MRM色谱图

## 3 结论

本文采用PSA+C_18_+GCB+EMR-Lipid混合填料分散固相萃取,采用氟化铵水溶液-甲醇体系作为流动相,建立了超高效液相色谱-串联质谱同时测定牛肉中17种PFASs的分析方法。方法不仅可以显著提高回收率,而且可以提高PFASs的检测灵敏度,具有操作简便、高效、准确、灵敏的特点,适用于牛肉样品中痕量PFASs的快速检测。
